# Tyrosine-Capped Pt Nanozyme Functionalized with cDNA: An Innovative Sensor Designed for One-Step Detection of the miRNA-21 Biomarker

**DOI:** 10.1021/acsomega.5c05564

**Published:** 2025-08-24

**Authors:** Sanam Garehbaghi, Lukas Richtera, Zeynep Altintas, Amir M. Ashrafi

**Affiliations:** † Central European Institute of Technology, 48274 Brno University of Technology, Brno 61200, Czech Republic; ‡ Department of Chemistry and Biochemistry, 309613 Mendel University in Brno, Brno 61300, Czech Republic; § Bioinspired Materials and Biosensor Technologies, Institute of Materials Science, Faculty of Engineering, 9179 Kiel University, Kiel 24143, Germany; ∥ Kiel Nano, Surface and Interface Science (KiNSIS), Kiel University institution, Kiel 24118, Germany; ⊥ Institute of Photonics and Electronics, 119450 Czech Academy of Sciences, Prague 18200, Czech Republic

## Abstract

The platinum nanozymes capped with tyrosine (Pt-Tyr NZ) were synthesized which demonstrated peroxidase and oxidase catalytic activities toward 3,3′,5,5′-tetramethylbenzidine (TMB). The kinetic analyses revealed that Pt-Tyr NZ exhibited higher catalytic activity and substrate affinity for both H_2_O_2_ and TMB compared to horseradish peroxidase (HRP), indicating its superior efficiency in biosensing applications. The synthesized Pt-Tyr NZ was reversibly conjugated with complementary DNA (cDNA), which can selectively hybridize with miRNA-21, a cancer biomarker, and after hybridization with miRNA-21, detaches from the surface of Pt-Tyr NZ. Upon cDNA conjugation, the catalytic activity of Pt-Tyr NZ decreased, but it was restored in the presence of miRNA-21, providing a one-step spectrophotometric biosensing method for miRNA-21 determination. Optimization of parameters affecting the sensitivity of the developed method for miRNA-21 detection was performed, including the tyrosine-to-platinum ratio in Pt-Tyr NZ synthesis, Pt-Tyr NZ concentration, and cDNA concentration for conjugation. The developed method was fully validated and showed promising results in terms of reproducibility (RSD = 1.26%, 95% confidence interval = 0.03, *n* = 6), repeatability (RSD = 0.83%, *n* = 5), and accuracy for spiked miRNA-21 determination in 10% filtered human serum samples (recovery rate = 109.7 ± 4.2%). It showed a linear dynamic range (LDR) from 37.1 to 185 nM, with a limit of detection (LOD) of 11.1 nM and a limit of quantification (LOQ) of 36.9 nM.

## Graphical abstract

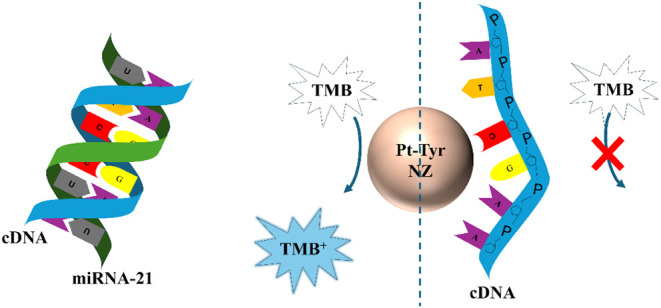

## 1 Introduction

MicroRNA-21 (miRNA-21) is a 22-nucleotide, noncoding RNA. Primary miRNA-21 is produced in the cell nucleus, converted into a premature form,^1^ and transferred to the cytoplasm, where it matures into single-stranded 22-nucleotide miRNA-21. The overexpression of miRNA-21 promotes cell division and replication while suppressing the expression of antitumor genes.^1–3^ In recent studies, the overexpression of miRNA-21 was observed in a variety of cancer types, such as pancreatic, colorectal, breast, colon, nonsmall cell lung, melanoma, endometrial, esophageal, renal, thyroid, and acute myeloid leukemia.^4^ Therefore, miRNA-21 present in human body fluids (e.g., blood serum and plasma) can be regarded as a highly stable and efficient biomarker for cancer diagnosis, even under harsh conditions. Additionally, quantification of miRNA-21 with high specificity enables the differentiation of various stages of a specific type of cancer, particularly when combined with other biomarkers.^2,5–7^ This is critically important for the medical sector addressing cancer, the second leading cause of death, as early-stage prognosis improves treatment effectiveness.^8^ The employment of conventional analytical techniques for miRNA-21 identification and quantification, such as quantitative real-time polymerase chain reaction (qRT-PCR),^9^ microarray analysis,^10^ and Northern blotting,^11^ is limited by their complex sample preparation, labor-intensive operation, time-consuming nature, and high cost.^12^ This has led to the growing development of biosensors for diagnosis due to their low cost and simple operation, promoting point-of-care (POC) application.

Among the numerous types of biosensors, optical and electrochemical biosensors have found wide application because of their simplicity, cost-effectiveness, robustness, and compact instrumentation.^13,14^ Affinity biosensors are commonly used for the detection and quantification of biomarkers, including proteins, nucleic acids, antigens, and antibodies. This approach involves using a capture (primary) probe that specifically binds to the target analyte (biomarker) and a signal (secondary) probe labeled with signal-generating tags.^15^ The secondary probe binds to the capture probe-analyte complex, producing an analytical signal that can be used for detection and quantification. Therefore, two probes and two incubation steps are required, which make this approach complicated.^16–18^ Label-free biosensors, such as impedimetric,^19^ surface plasmon resonance,^20^ and quartz crystal microbalance^21^ biosensors, function based on the specific binding of the capture probe and the analyte and are less complicated and time-demanding. However, nonspecific adsorption in these types of biosensors causes inaccurate results and reduces reliability.^22^

The advancements in nanomaterials research have benefited biosensing, where nanomaterials can play several roles in biosensing, such as labels and carriers for the capture probe.^23^ A novel approach in affinity biosensors is the employment of nanomaterials with enzyme-like activity, called nanozymes (NZs), whose catalytic activity is deactivated by immobilization of the capture probes and restored upon detachment of the capture probes after binding with the target analyte.^24–27^ This process generates a measurable analytical signal in the presence of the corresponding substrate of the employed NZ.^28,29^ In this approach, only one capture probe is required, which binds with the analyte in one step. The effectiveness of this approach depends on the preparation of NZ with high catalytic activity, as well as the proper immobilization of the capture probe on the NZ surface, which should be easily detached in the presence of the target analyte.^30^

In this work, the one-step determination of miRNA-21 was aimed. Pt-Tyr NZ with peroxidase-like activity was prepared and reversibly conjugated with a capture DNA (cDNA) complementary to miRNA-21, suppressing the catalytic activity toward oxidation of TMB in the presence of H_2_O_2_. Upon introduction of miRNA-21, the cDNA hybridizes with its target and detaches from the Pt-Tyr NZ surface, restoring catalytic activity and generating a measurable analytical signal (Scheme 1).

**Scheme 1:**
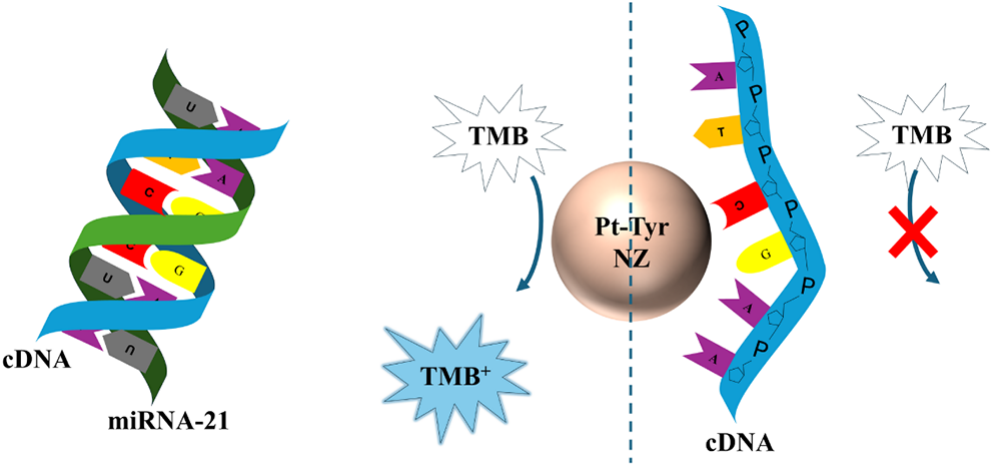
cDNA-Functionalized Pt-Tyr NZ with Passivated Catalytic Activity and Reversible Catalytic Recovery in the Presence of miRNA-21

## 2 Materials and Methods

### 2.1 Reagents, Chemicals, and Oligonucleotides

The dialysis tubing cellulose membrane, with an average flat width of 43 mm, an inner diameter of 1.7 mm, and a molecular weight cutoff of 14 kDa, hexachloroplatinic­(IV) acid hexahydrate (H_2_[PtCl_6_]·6H_2_O), l-tyrosine, 3,3′,5,5′-tetramethylbenzidine dihydrochloride hydrate (TMB), glacial acetic acid, sulfuric acid (H_2_SO_4_), sodium borohydride (Na­[BH_4_]), hydrogen peroxide (H_2_O_2_, 30% w/w), potassium hexacyanoferrate­(II) trihydrate (K_4_[Fe­(CN)_6_]·3H_2_O), methylene blue, and salt-free lyophilized powder of horseradish peroxidase (HRP, type VI-A, EC 1.11.1.7) were purchased from Sigma-Aldrich Chemical Co. (St. Louis, MO, USA). NaOH, HCl, NaCl, KCl, Na_2_HPO_4_, and KH_2_PO_4_ all in ASC grade from the same supplier, were used for the preparation of 0.1 M phosphate-buffered saline (PBS), pH 7.4, in nuclease-free molecular biology grade (MBG) water (VWR, Radnor, PA, USA). The HRP enzyme was dissolved in PBS solution for the kinetic assay experiment.^31^

A reverse-osmosis apparatus (Aqual 25, Ceska, Czech Republic) was utilized to provide demineralized water before being treated using a Millipore system (Billerica, MA, USA) to obtain ultrapure water with a resistivity of 18.20 MΩ·cm. The synthesized cDNA, miRNA-21, and lung-specific miRNAs such as mir-17–3p and mir-1268b (Table S1) in HPLC grade, as well as human serum (HS), were also purchased from Sigma-Aldrich Chemical Co.

### 2.2 Synthesis of Tyrosine (Tyr)-Capped Pt NZ

Pt-Tyr NZ was synthesized following the procedure described by Li *et al.*,^32^ with slight modifications. In the original synthesis by Li *et al*., polyvinylpyrrolidone (PVP)-capped Pt NZ was prepared in 50 mL of aqueous solution containing 1 mM H_2_[PtCl_6_]·6H_2_O and 20 mg of PVP as the capping agent. In another study by Wang et al., Tyr-capped Au NZ with reversible cDNA conjugation properties was synthesized using 5 mg of Tyr in an equivalent 50 mL aqueous solution of 1 mM H­[AuCl_4_]·4H_2_O.^33^ Based on these studies, in this work, 50 mL of ultrapure water containing 1 mM H_2_[PtCl_6_]·6H_2_O was mixed with Tyr at concentrations equivalent to the previous studies and three intermediate concentrations (0.00, 5.00, 10.0, 15.0, and 20.0 mg). Subsequently, 0.5 mL of 100 mM NaBH_4_ was rapidly added as a reducing agent under stirring (600 rpm), and the mixture was stirred for 10 h at ambient temperature. The optimum mass of 15.0 mg Tyr-capped Pt NZ was selected based on scanning electron microscopy (SEM) results in Figure S1a–e and was dialyzed overnight in ultrapure water in order to remove the unreacted ions and free Tyr and was labeled as Pt-Tyr NZ.^33^ The concentration of the prepared Pt-Tyr NZ dispersion in water was 0.19 ± 0.01 g L^–1^ that was determined by evaporating 3 mL solutions at room temperature for 1 week to reach a constant mass and weighing in four repeats.

### 2.3 Characterization of Pt-Tyr NZ

Transmission electron microscopy (TEM) images of Pt NZ samples without Tyr capping (Figure 1a) and with 15.0 mg of Tyr capping (Figure 1b) were obtained using the instrument (JEM-2010, JEOL Ltd., Tokyo, Japan), operated at an accelerating voltage of 160 kV.

**Figure 1:**
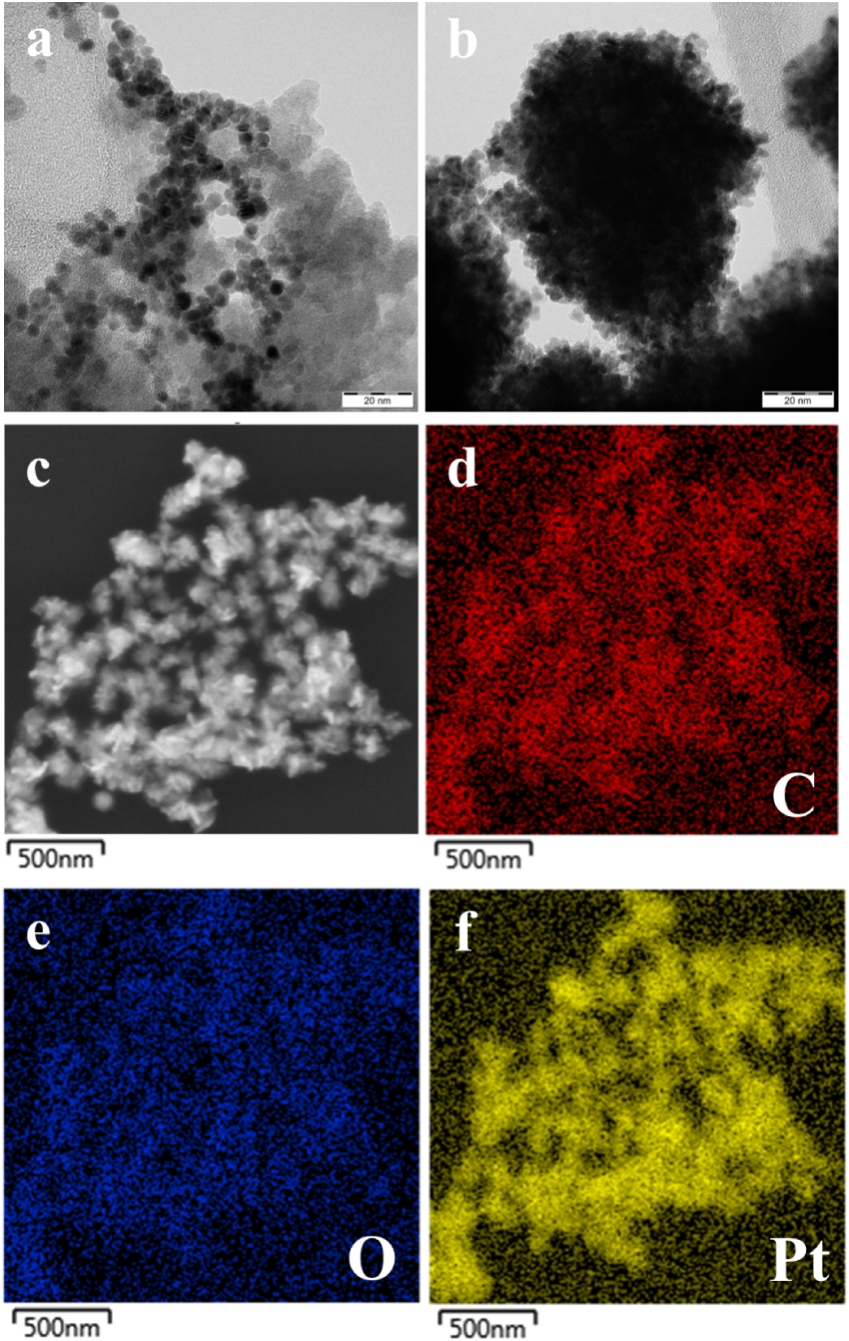
TEM images for Pt NZ without Tyr modification (a) and for Pt-Tyr NZ with 15.0 mg of Tyr (b) in the synthesis solution. SEM images for Pt-Tyr NZ (c) with EDX mapping for the synthesized Pt-Tyr NZ (d–f).

A MAIA 3 SEM (TESCAN Ltd., Brno, Czech Republic), equipped with a field emission gun (FEG; TESCAN Ltd., Brno, Czech Republic) and using an in-beam secondary electron detector at working distances between 2.90 and 2.99 mm, was employed to assess the morphology of the Pt NZ with different amounts of Tyr capping (0.00, 5.00, 10.0, 15.0, and 20.0 mg), as well as cDNA-functionalized Pt-Tyr NZ. For the elemental mapping, a MIRA 2 SEM under high-vacuum conditions was employed with an energy-dispersive X-ray spectroscopy (EDX) detector, X-MAX 50 (Oxford Instruments plc, Abingdon, UK), at an accelerating voltage of 15 kV and a working distance of 15 mm. ImageJ (Version 1.54 g, National Institutes of Health, Bethesda, MD, USA) software was utilized for size distribution analysis, while OriginPro (version 2024, OriginLab Corporation, Northampton, MA, USA) software was used for applying a Gaussian fit for size distribution analysis.

FTIR spectra were acquired using a Bruker INVENIO-R FTIR spectrometer (Bruker Optics Inc., Billerica, MA, USA) equipped with a single-reflection diamond attenuated total reflectance (ATR) accessory. To ensure full contact between the sample and the diamond ATR, a fixed load was applied to a small quantity of the sample, which was analyzed directly in solid form. Background spectra were collected prior to each measurement. The spectra were recorded at 25 °C with a resolution of 2 cm^–1^, where a total of 128 interferograms were combined to obtain each spectrum, with six repeats of measurement. Bruker OPUS version 8.1 software package (Bruker Optics Inc., Billerica, MA, USA) was used for spectral acquisition, and JDXview version 0.2 software was utilized for additional spectral evaluation.

The zeta (ζ)-potential was measured using a Zetasizer Nano ZS instrument (Malvern Instruments Ltd., Worcestershire, UK). Each sample measurement was performed in triplicate at a concentration of about 0.01 g L^–1^ of the Pt-Tyr NZ at 25 °C in disposable DTS1070 cuvettes (Malvern Panalytical Inc., Westborough, MA, USA). The ζ-potential was measured with an equilibration time of 120 s, and calculations were based on the Smoluchowski model, using a diminishing particle concentration and a Henry function (*F*(κ*a*)) value of 1.50.

### 2.4 Catalytic Activity Determination

An Infinite M200 Pro plate reader (Tecan, Männedorf, Switzerland), equipped with Tecan i-control software, was used to record absorbance spectra and absorbance at a specific wavenumber, which was used for the calculation of the catalytic activity of Pt NZ on a transparent 96-well, nontreated surface plate (Nunc, Thermo Fisher Scientific, Waltham, MA, USA) at 25 °C.

## 3 Results and Discussion

### 3.1 Characterization Study of Pt-Tyr NZ

The TEM images (Figure 1a,b) reveal that Tyr plays a crucial role in the controlled synthesis of Pt NZ, significantly influencing its morphology, size, and dispersion. In the absence of Tyr, the Pt NZ exhibits irregular aggregation of very small particles with a mean particle size of 3.6 ± 0.4 nm (Figure 1a). Conversely, Pt-Tyr NZ consists of semispherical-shaped particles with a particle size of ∼50 nm, formed as an aggregation of very small-sized particles, suggesting that Tyr acts as a stabilizing and capping agent (Figure 1b). Notably, increasing the concentration of Tyr during the synthesis results in a progressive increase in the particle size of Pt-Tyr NZ.^32^ The SEM image (Figure 1c) with related EDX elemental mapping (Figure 1d) of the prepared Pt-Tyr NZ confirmed the presence of carbon (C), oxygen (O), and Pt, indicating successful synthesis of Pt NZ and surface stabilization with Tyr molecules.

Pt NZ synthesized in the absence of Tyr as a stabilizing or capping agent (Figure S1a) appears highly aggregated, forming nanoclusters with estimated sizes below 10 nm. In Pt NZ synthesized with 5.00 mg Tyr (Figure S1b), particles grow to a mean particle size of 35 ± 8 nm with irregular growth shapes and are non-monodispere. The particles exhibit a more defined structure but still lack uniformity in size and shape. Pt NZ synthesized with 10.0 mg of Tyr (Figure S1c) displays enhanced monodispersity with a mean particle size of 46 ± 16 nm. Further increasing the Tyr amount to 15.0 mg (Figure S1d) in the synthesis medium results in well-separated, semispherical Pt NZ with uniform size distribution. This indicates that Tyr effectively controls nucleation and growth processes, leading to a stable colloidal dispersion with a mean particle size of 52 ± 7 nm. At the highest Tyr amount of 20.0 mg (Figure S1e), Pt NZ exhibits an increase in particle size, reaching a mean particle size of 128 ± 5 nm. Excess Tyr may influence the surface interactions, possibly leading to overstabilization or altered growth kinetics. Pt-Tyr NZ functionalization with cDNA (Figure S1f) shows a similar particle size and shape as nonfunctionalized Pt-Tyr NZ (Figure S1d) with mean particle size of 52 ± 11 nm. The SEM results highlight the crucial role of Tyr as a capping and stabilizing agent in Pt NZ synthesis. Increasing Tyr concentration enhances Pt NZ dispersion and uniformity, with the optimal amount observed at 15.0 mg. These findings suggest that controlled tuning of Tyr concentration is the key to obtaining well-defined Pt NZ with uniform morphological characteristics. This effect is likely due to the interaction of Tyr’s hydroxyl (−OH) group with Pt (IV) ions, which regulates nucleation, prevents excessive particle fusion, and enhances surface stability.^34^ The improved dispersion of Pt NZ in the presence of Tyr is expected to enhance its catalytic and sensing performance by increasing surface area and functional group availability. Further, Tyr can provide a green synthesis procedure by increasing the biocompatibility of Pt NZ for biosensing applications. Tyr can also have an effect on Pt NZ formation as a reducing agent.^35^ However, further increasing the Tyr concentration to 20.0 mg leads to significant agglomeration of the Pt NZ (Figure S1e).

The ζ-potential of Pt NZ before dialysis was measured to be −37.1 ± 10.5 mV (Figure S2a), indicating high colloidal stability due to strong electrostatic repulsion between particles, which helps prevent aggregation.^34^ After dialysis, Pt-Tyr NZ loses a portion of surface-bound Tyr, resulting in a significant reduction in surface charge, with the ζ-potential decreasing to −8.93 ± 5.23 mV (Figure S2b). The conjugation of Pt-Tyr NZ with cDNA introduces additional negative charge due to the negatively charged phosphate backbone of DNA, resulting in a ζ-potential of −51.8 ± 12.5 mV (Figure S2c).^30^ Upon hybridization with miRNA-21, a portion of the surface-bound cDNA is displaced from the Pt-Tyr NZ, leading to a reduction in surface negative charge. As a result, the ζ-potential becomes less negative, shifting to −26.3 ± 7.9 mV (Figure S2d), confirming the interaction between cDNA and miRNA-21.

The FTIR spectrum (Figure 2) exhibits distinct peaks at 1606/1580 cm^–1^ and 1365/1332 cm^–1^, corresponding to the asymmetric and symmetric stretching vibrations of the C=O bond in the carboxylic functional groups of Tyr, respectively. Peaks at 1514 cm^–1^ and 1243 cm^–1^ are attributed to the C=C stretching vibrations within the benzene ring of Tyr and the C–O stretching vibrations of the phenolic group in Tyr, respectively.^36^ The N–H stretching or O–H stretching vibrations in the Tyr molecule are presented as a broad band in the range of 2500–3300 cm^–1^.^37^ For the Pt NZ spectra without Tyr, no significant peaks are observed, except for a weak peak at 637 cm^–1^, which can be assigned to the Pt–O bond due to oxidized Pt species present on the surface of Pt NZ.^38^ However, the spectrum of Pt-Tyr NZ reveals a broad peak at 1630 cm^–1^ that is absent in the Pt NZ spectrum. This peak is attributed to the C=O stretching vibrations, indicating the stabilization of Tyr on the surface of Pt-Tyr NZ through carbonyl groups.^36^ Additionally, a broad peak in the range of 3000–3600 cm^–1^ is observed in Pt- Tyr NZ, which corresponds to the O–H and N–H stretching vibrations of Tyr. This further supports the successful surface modification of the prepared Pt-Tyr NZ with Tyr.

**Figure 2:**
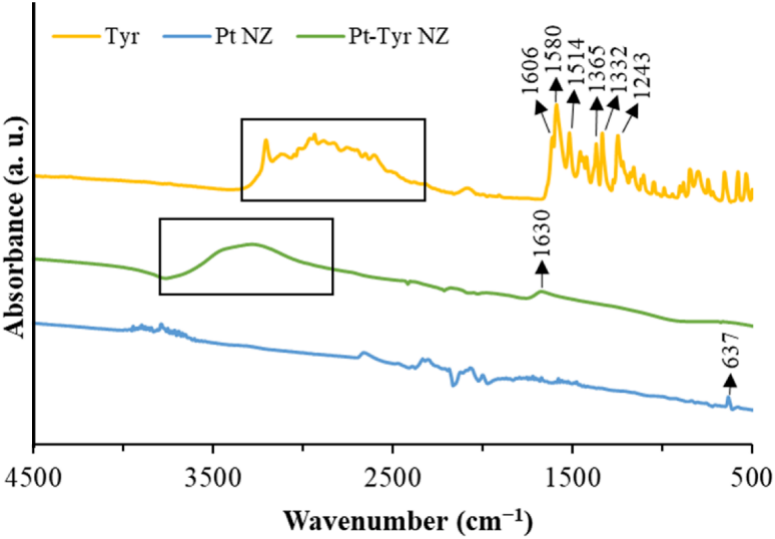
ATR-FTIR spectra of Tyr, Pt NZ, and Pt-Tyr NZ.

### 3.2 Catalytic Activity of Pt-Tyr NZ

The peroxidase and oxidase catalytic activities of Pt-Tyr NZ were investigated toward a TMB substrate, which is colorless in its reduced form. The oxidation of TMB in the presence of Pt-Tyr NZ was spectrophotometrically assessed in a solution containing Pt-Tyr NZ and TMB. In a one-electron step oxidation of TMB, TMB^+^ was formed, with a blue color and an absorbance peak at 650 nm.^39^ The blue color in TMB^+^ is due to the formation of an electron transfer complex between one TMB molecule and the TMB^●+^ radical cation.^40^

The absorbance values of nearly zero at 650 nm for TMB + H_2_O_2_, Pt-Tyr NZ, and Pt-Tyr NZ + H_2_O_2_ at the same concentrations were observed (Figure 3a). The appearance of a peak at 650 nm for Pt-Tyr NZ + TMB and Pt-Tyr NZ + TMB + H_2_O_2_ samples can be attributed to the oxidase and peroxidase activities of Pt-Tyr NZs, respectively, which convert TMB to TMB^+^ (Figure 3a). Over time, the oxidase and peroxidase activities of Pt-Tyr NZ increase, while Pt-Tyr NZ, Pt-Tyr NZ + H_2_O_2_, and TMB + H_2_O_2_ maintain absorbance values close to zero (Figure 3b). The higher absorbance intensity of Pt-Tyr NZ + TMB + H_2_O_2_ compared to Pt-Tyr NZ + TMB indicates that Pt-Tyr NZ exhibits stronger peroxidase-like activity than oxidase activity, suggesting that utilizing its peroxidase activity could enhance biosensing sensitivity.

**Figure 3:**
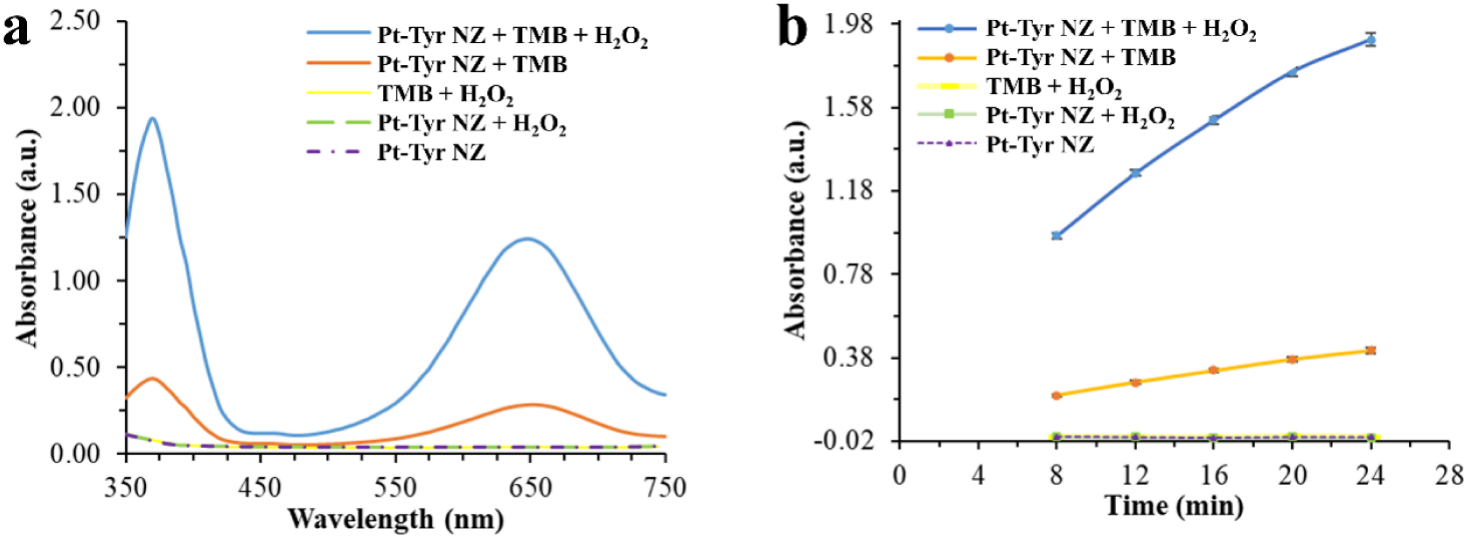
Absorbance spectra scanned after 8 min of reaction time (a) and absorbance values at 650 nm vs 500 nm over an 8–24 min measurement (b) for different media of Pt-Tyr NZ + TMB + H_2_O_2_, Pt-Tyr NZ + TMB, TMB + H_2_O_2_, Pt-Tyr NZ + H_2_O_2_, and Pt-Tyr NZ.

The production of blue-colored TMB^+^ proceeds continuously and is measured right after 8 min of peroxidase reaction time, resulting in peak maxima at a 650 nm wavelength. When the stop solution is added right after 8 min of peroxidase reaction time, TMB^+^ is fully oxidized to TMB^2+^ (the two-electron oxidation form of TMB) in a short period of time, resulting in a yellow color. This causes a blue shift in the peak maxima to a 450 nm wavelength, which is sharper and has higher peak intensity compared to the TMB^+^ peak (Figure 4a). Screening the absorbance intensity of the produced TMB^+^ peak further after 8 min until 24 min of peroxidase reaction shows an increase in absorbance intensity, while the absorbance intensity of TMB^2+^ after adding the stop solution no longer changes after 8 min of peroxidase reaction, even with prolonged time until 26 min, proving that the addition of a stop solution provides a stable platform for biosensing (Figure 4b).^40,41^

**Figure 4:**
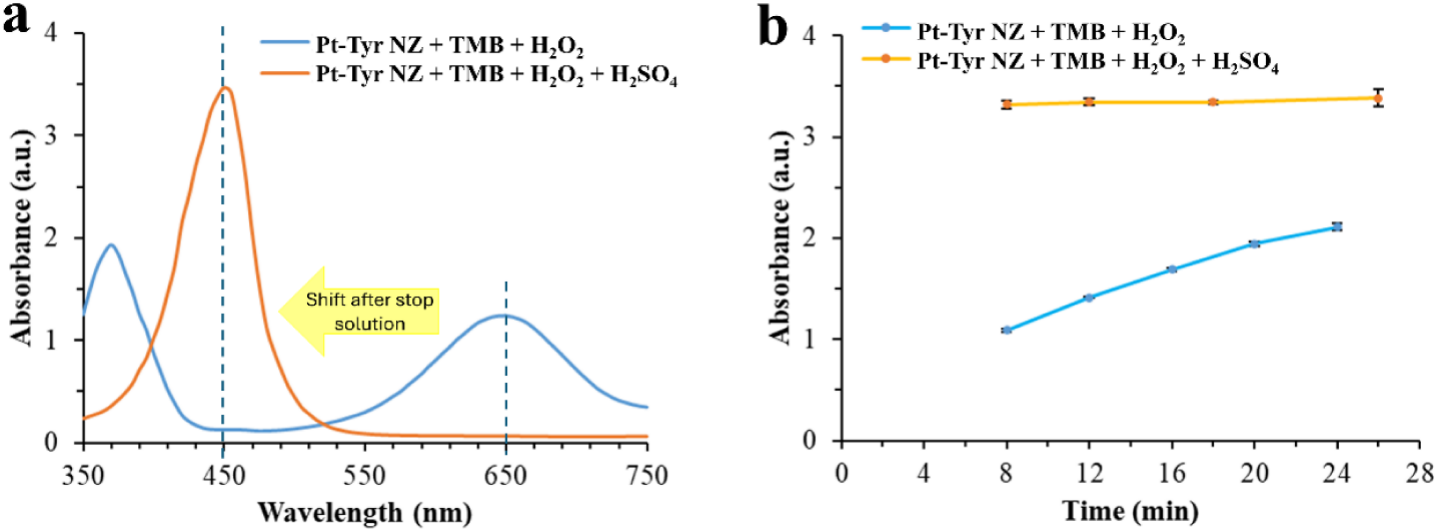
Absorbance spectra (a) for peroxidase activity of Pt-Tyr NZ at an incubation time of 8 min for solutions with (orange) and without the addition of stop solution (blue). Time-dependent absorbance intensity change (8–26 min) (b) of Pt-Tyr NZ + TMB + H_2_O_2_, for solutions with (orange) and without (blue) the addition of stop solution.

The addition of the stop solution 0.5 min after the start of the peroxidase reaction produces only a small amount of TMB^2+^ while adding the stop solution after a longer catalytic reaction time produces a higher amount of TMB^2+^. The increase in absorbance intensity over time can occur because more TMB^+^ is produced and is available for conversion to TMB^2+^. The absorbance intensity of TMB^2+^ increases sharply until 8 min and then continues to rise at a slower rate, suggesting that the addition of a stop solution after 8 min can provide a suitably high signal for biosensing means (Figure S3).

The Michaelis–Menten (MM) curves^42^ show that the reaction rate (ν) for Pt-Tyr NZ and HRP gradually increased with increasing H_2_O_2_ concentration. However, the ν magnitude declined at higher concentrations of H_2_O_2_ (Figure S4a,c). The same behavior was observed for TMB as the substrate, where a gradual increase in ν magnitude was observed with increasing TMB concentration, followed by a decline at higher concentrations of TMB (Figure S5a,c). To compare the catalytic performance of Pt-Tyr NZ relative to the widely used HRP, their kinetic parameters, *K*_M_, *V*_max_, and *k*_cat_, were analyzed using their Lineweaver–Burk plots (Table 1) for TMB (Figure S4b,d) and H_2_O_2_ (Figure S5b,d) for Pt-Tyr NZ and HRP, respectively. Pt-Tyr NZ exhibits a significantly higher *k*_cat_ compared to HRP, being 8 and 10 times greater toward H_2_O_2_ and TMB, respectively. This indicates that the Pt-Tyr NZ can catalyze the reaction at a much faster rate than HRP when fully saturated with substrate. The drastic increase in *k*_cat_ suggests that the catalytic sites on Pt-Tyr NZ possess superior intrinsic peroxidase activity, possibly due to enhanced electron transfer properties or the presence of more active sites than an enzyme, which has only one active site.^43^ The *K*_M_ value of Pt-Tyr NZ is 0.56 and 0.28 times that of HRP toward H_2_O_2_ and TMB, respectively. This suggests that Pt-Tyr NZ has a higher affinity for the substrate compared to HRP. The catalytic efficiency can be determined by comparing the *k*_cat_/*K*_M_ value of Pt-Tyr NZ and HRP, which for Pt-Tyr NZ is 14 and 38 times higher than HRP toward H_2_O_2_ and TMB, respectively. This substantial catalytic efficiency in Pt-Tyr NZ with respect to HRP can be attributed to the higher electron transfer properties of Pt-Tyr NZ.^44,45^ Also, using a Tyr capping agent, through interaction with TMB, can notably increase the catalytic activity toward TMB.^46^ These findings demonstrate that Pt-Tyr NZ is a promising alternative to the natural HRP enzyme for biosensing applications because of its catalytic reaction with a superior reaction rate, excellent substrate affinity, and high catalytic efficiency.

**Table 1:** Kinetic Parameters of Pt-Tyr NZ and HRP toward H_2_O_2_ and TMB

				Kinetic parameters		
Catalyst type	Catalyst concentration (mg L^–1^)	Substrate	*K*_M_ (mM)	*V*_max_ (μM min^–1^)	∼*k*_cat_(s^–1^) × 10^3^	∼*k*_cat_/*K*_M_(mM^–1^ s^–1^) × 10^3^
HRP	1.00 × 10^–3^	H_2_O_2_	1.14	7.99	4.69	4.11
Pt-Tyr NZ	2.50	H_2_O_2_	0.643	5.02	37.3	58.1
HRP	4.00 × 10^–3^	TMB	0.497	16.8	3.08	6.19
Pt-Tyr NZ	2.00	TMB	0.137	4.32	32.3	236

Increasing the concentration of Pt-Tyr NZ to 2.00 mg L^–1^ resulted in a linear increase in absorbance intensity at 450 nm with a minimal standard deviation. However, at higher concentrations, the absorbance intensity decreased, accompanied by an increase in standard deviation. This phenomenon can be attributed to the agglomeration of Pt-Tyr NZ at higher concentrations, leading to enhanced light scattering, which reduces the absorbance signal from TMB^2+^ (Figure 5).^47^

**Figure 5:**
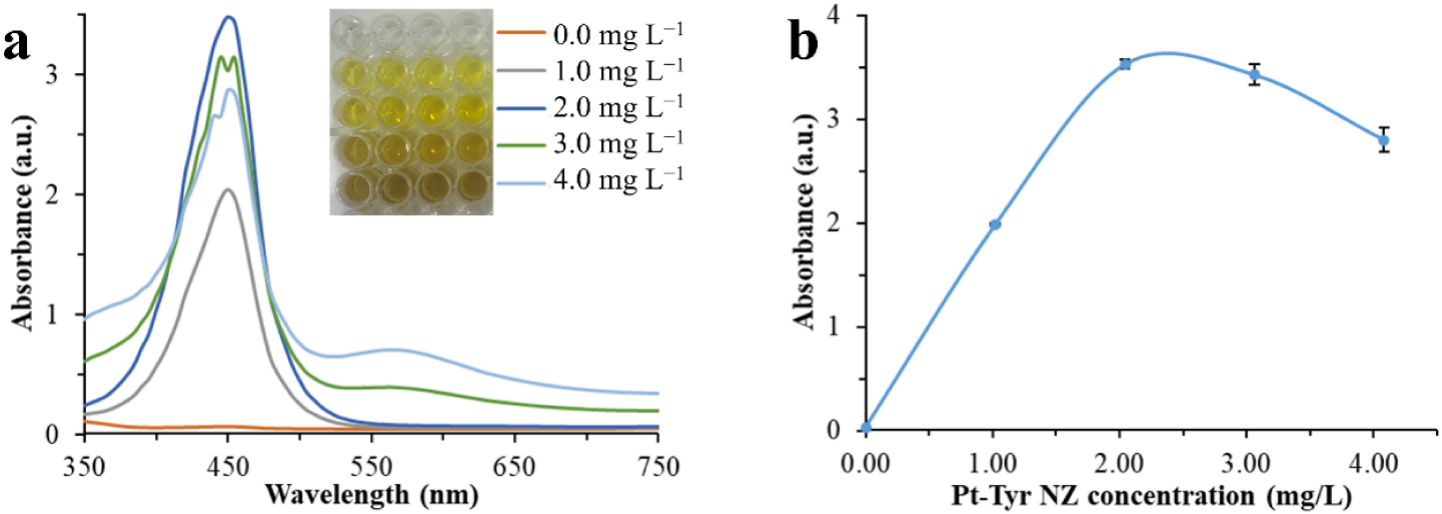
Effect of concentration changes of Pt-Tyr NZ with absorbance spectra (a) and absorbance response (b) in the substrate solution on absorbance intensity at 450 nm after adding the stop solution, 8 min following the TMB^+^ reaction. The inset shows a color change for different concentrations of Pt-Tyr NZ after adding the stop solution.

### 3.3 cDNA Regulation of Peroxidase Activity of Pt-Tyr NZ

The cDNA interacts with the surface of Pt-Tyr NZ and passivates its active surface, decreasing the catalytic activity and the production of TMB^2+^ content. Peroxidase activity significantly decreased with the increasing concentration of cDNA from 0 to 556 nM, where 39% of catalytic activity was passivated (Figure 6). This is because Pt-Tyr NZ has an affinity toward amine groups in cDNA.^46,48^ The Tyr-stabilized Pt NZ can provide better biocompatibility and prevent denaturation of cDNA, while providing better adsorption for cDNA through hydrogen bonding and π–π stacking on the Pt-Tyr NZ surface.^49^ The negative surface charge of Pt-Tyr NZ indicates that cDNA is not adsorbed onto its surface via electrostatic interactions, due to the repulsion between the negatively charged phosphate backbone of cDNA and the negatively charged Pt-Tyr NZ.^50,51^ A slight reduction in the peroxidase activity was subsequently found with the further increase in the concentration of cDNA from 556 nM to 927 nM (Figure 6).

**Figure 6:**
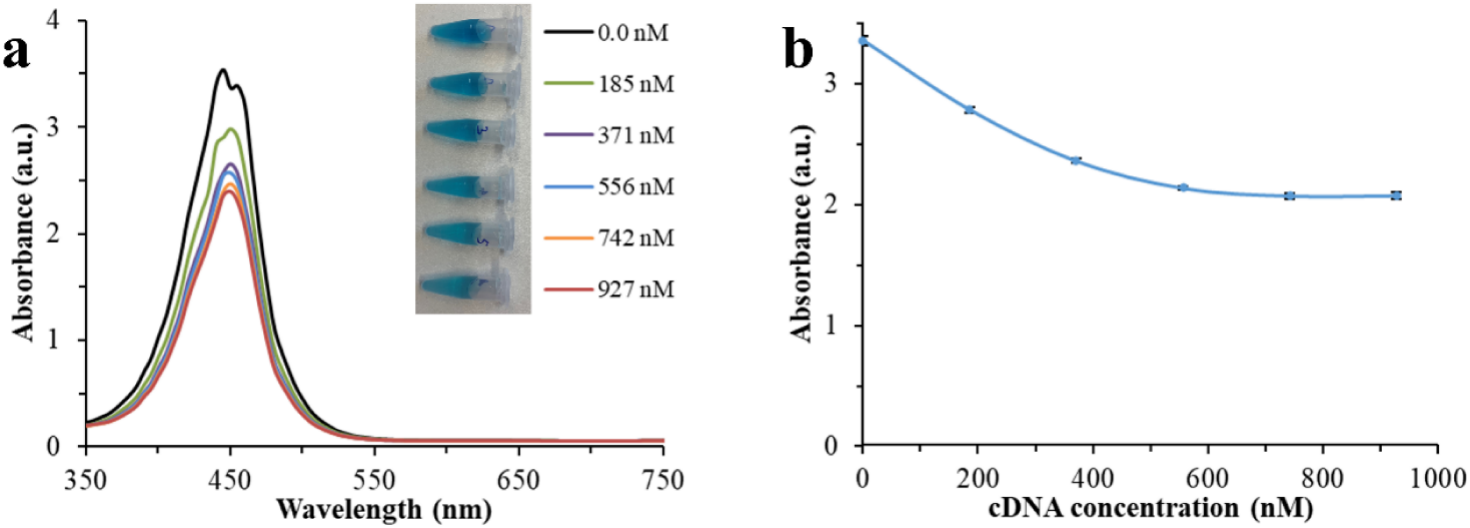
Absorbance spectra (a) and absorbance response (b) for peroxidase activity of 2.00 mg L^–1^ Pt-Tyr NZ as a function of cDNA concentration after adding the stop solution, 8 min following the TMB^+^ reaction. The inset shows a color change for different concentrations of cDNA before adding the stop solution.

A concentration of 556 nM of cDNA was used to interact with 2.00 mg L^–1^ Pt-Tyr NZ, for further studies in the detection of miRNA-21 in Sections 3.4–3.6. As provided in Table 2, surface modification on transducers or probes can be carried out to induce functional groups such as NH_2_, SH, COOH, and poly­(methyl methacrylate) to provide covalent bonding for biosensing strategies in miRNA-21 detection. However, this necessitates a prolonged incubation time.^30,52^ Other strategies in Table 2 utilize modifications such as His-tag and streptavidin–biotin for affinity interactions, which are reversible interactions as well. However, the dissociation of these affinity interactions is not as readily achieved as with hydrogen bonding or π–π stacking, limiting their suitability for one-step biosensing methods.^53^ Surface passivation or blocking of the transducer, using self-assembled monolayers or bovine serum albumin, is another approach to minimizing nonspecific adsorption. However, it adds additional steps to the sensor preparation process.^52,53^

**Table 2:** Previous Biosensor Studies on miRNA-21 Detection

Nanomaterials	Surface modifications	Number of enzymes	Capture DNA (cDNA) modifications	Number of probes	Detection steps	Sensor type	LDR (nM)	LOD (nM)	Detection time (min)	Ref
-	PMMA^a^, neutravidin		dual biotin	3	1. miRNA-21	fluorescent	5.00 × 10^–2^–50.0	2.10 × 10^–1^	1440	^56^
					2. cDNA-miRNA hybrid antibody					
					3. secondary antibody					
Ru(bpy)_3_^2+^@MOF^a^	BSA^a^, streptavidin, −COOH		biotin, NH_2_	2	1. miRNA-21	electrochemiluminescent	1.56–100	1.44	90	^53^
					2. Ru(bpy)_3_^2+^@MOF@pDNA					
Graphene/Au NP^a^	-	1	peptide nucleic acid	1	1. miRNA-21	spectrophotometry	10.0–980	3.20	94	^57^
					2. 16 cycle PCR					
-	-	5	-	3	1. miRNA-21 T	personal glucose meter	5.00–150	3.65	110	^58^
					2. exonuclease					
					3. cascade of 4 enzymes					
-	MCH^a^	0	SH, methylene blue	1	1. miRNA-21	electrochemical square-wave voltammetry	70.0–345	34.0	20	^52^
-	His-tag streptavidin	1	biotinyl	2	1. miRNA-21	electrochemical amperometric	3.00–100	9.10 × 10^–1^	120	^59^
					2. zinc finger protein					
					3. HRP					
-	-	1	apurinic/apyrimidinic, 6-FAM, Dabcyl	2	1. miRNA-21	fluorescent	2.50–40.0	2.50 × 10^–1^	75	^60^
Au NP^a^	streptavidin	1	Texas Red, biotin, SH	4	1. miRNA-21	fluorescent	0.120–1.20	1.80 × 10^–2^	150	^61^
					2. exonuclease III					
					3. DNA-b + Au NP					
					4. DNA-d					
					5. DNA-c					
Pt-Tyr NZ	-	0	-	1	1. miRNA-21	spectrophotometry	37.1–185	11.1	36.9	this study

a Ru­(bpy)_3_^2+^@MOF: zinc oxalate metal–organic framework, PCR: polymerase chain reaction, PMMA: poly­(methyl methacrylate), BSA: bovine serum albumin, MCH: 6-mercapto-1-hexanol, and Au NP: Au nanoparticle.

### 3.4 Spectrophotometry Assay for miRNA-21 Detection

The increased level of miRNA-21 increases the release rate of cDNA from the surface of Pt-Tyr NZ, leading to a highly restored active site and the recovery of peroxidase activity (Figure 7a,b).^33^ This occurs because the interaction between cDNA and the surface of Pt-Tyr NZ is a weak noncovalent type, while the interaction between cDNA and miRNA is stronger.^43^ The presence of miRNA-21 competes with the surface of Pt-Tyr NZ in binding with cDNA and detaches cDNA from the surface of Pt-Tyr NZ to hybridize with miRNA-21. Thus, cDNA conjugation with Pt-Tyr NZ is reversible in the presence of miRNA-21.^54^ Further, a hybrid cDNA-miRNA-21 has a more rigid structure compared to single-stranded cDNA, which decreases the effective interaction with the surface of Pt-Tyr NZ.^55^ Additionally, a hybrid ssDNA-miRNA-21 exposes its phosphate backbone, providing a more negative charge than ssDNA and allowing electrostatic repulsion, thereby detaching ssDNA-miRNA-21 from the surface of Pt-Tyr NZ, which has a negative ζ-potential.^50,51^ The calibration curve for miRNA-21 shows an LDR of 0 to 185 nM, with a limit of detection (LOD) of 11.1 nM and a limit of quantification (LOQ) of 36.9 nM. The RSD for repeatability and reproducibility were 0.83% and 1.26%, respectively, and the reproducibility confidence interval was 0.03 at a 95% confidence level. Some miRNA-21 biosensors with corresponding linear dynamic range (LDR) and LOD values close to this work are listed in Table 2. Table 2 provides information on the synthesized nanoparticles, surface modifications, DNA probe functionalization, and the number of probes and enzymes used in biosensor development.^52,53,56–61^ Furthermore, the number of steps and incubation time required for detection after adding miRNA-21 are listed.^52,53,56–61^ For miRNA-21 detection, DNA probes were strategically modified to enable covalent bonding (e.g., SH, NH_2_, COOH), avidin–biotin interactions, fluorescent labeling (e.g., 6-FAM, Dabcyl, Texas Red), and electroactive tagging (e.g., methylene blue). Moreover, DNA probes with backbone modifications, such as peptide nucleic acid, or base modifications, such as apurinic/apyrimidinic sites, were used in sensing strategies. These DNA modifications increase the cost of sensor development and reduce the overall simplicity of the method. Similarly, an increased number of probs, and the use of enzymes such as exonuclease III, exonuclease T, polymerase, HRP, and apurinic/apyrimidinic endonuclease in these studies increase the cost and steps of miRNA-21 detection.^52,53,56–61^ In contrast, the miRNA-21 biosensor developed in this study requires minimal modification, fewer probes, enzymes, and detection steps compared to previous studies, enabling one-probe, one-step detection of miRNA-21.

**Figure 7:**
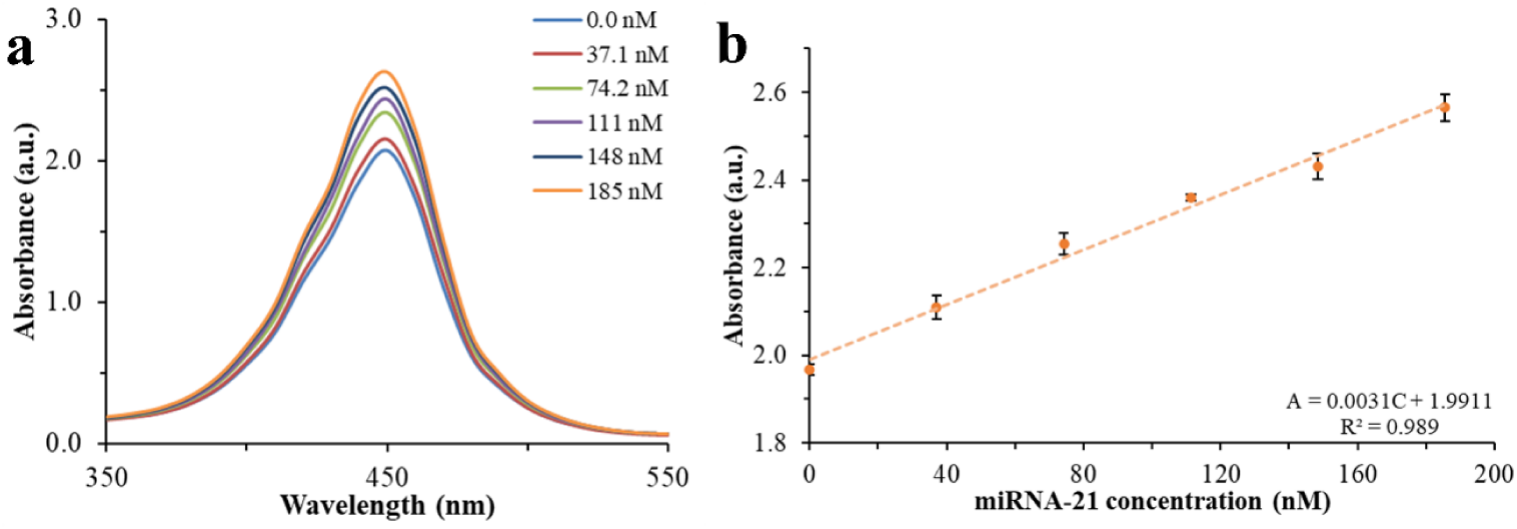
Absorbance spectra (a) and the standard calibration curve of spectrophotometric measurements for different concentrations of miRNA-21. A 2.00 mg L^–1^ Pt-Tyr NZ modified with 556 nM cDNA in the substrate solution was used, and the stop solution was added 8 min after the TMB^+^ reaction.

### 3.5 Selectivity Study

A selectivity study was performed in the presence of RNAs recognized as cancer biomarkers, and the results show that these RNAs have an insignificant effect on restoring the catalytic activity of Pt-Tyr NZ conjugated with cDNA compared to miRNA-21. This is due to the inability of non-target RNAs to hybridize with cDNA, causing its detachment from the surface of the Pt-Tyr NZ (Figure 8).

**Figure 8:**
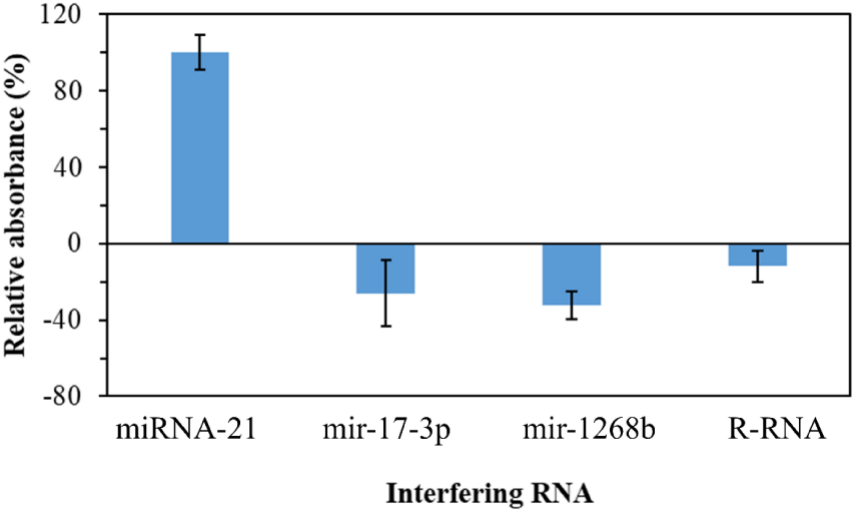
Selectivity study for the Pt-Tyr NZ-conjugated cDNA biosensing system by relative absorbance response for 74.2 nM of miRNA-21, mir-17–3p, mir-1268b, and R-RNA. Absorbance intensity was read at a wavelength of 450 nm versus 550 nm.

### 3.6 Real Sample Analysis

The potential use of the developed method in real sample analysis was tested in a filtered HS diluted 10% in 0.05 M PBS (10% FHS), and an LDR from 37.1 to 148 nM for miRNA-21 was obtained. After spiking the 10% FHS sample with 37.1 nM of miRNA-21, the content of miRNA-21 was determined by the developed method to be 40.7 nM, with a recovery rate of 109.7 ± 4.2%. (Figure S6). The presence of proteins in 10% FHS sample can lead to absorbance due to non-specific adsorption on the surface of Pt-Tyr NZ, decreasing the catalytic activity, which provides a similar amount of absorbance response over a longer period of time (16 min) compared to the PBS buffer. The developed biosensor, with a LDR concentration in the order of nM, can be applied in future studies for cancer diagnosis by differentiating the concentration of detected miRNA-21 in HS samples from healthy control and cancer patients.^58,62^ Additionally, the q-PCR technique can verify that the concentration of miRNA-21 in HS samples is in the fM range, and the miRNA-21 concentration in cancer patients reaches approximately 5 to 10 times higher than that in healthy controls, allowing differentiation between cancer patients and healthy controls.^63–66^ Therefore, to detect miRNA-21 at fM concentrations using the developed biosensor, a preconcentration step for miRNA-21 may be required.^19^

## 4 Conclusion

In this study, the synthesis, characterization, and biosensing application of Pt-Tyr NZ were investigated. The catalytic activities of the synthesized Pt-Tyr NZ toward TMB, including both peroxidase-like and oxidase-like functions, were evaluated. Results demonstrated that Pt-Tyr NZ exhibited significantly higher peroxidase activity compared to its oxidase activity. Kinetic analyses using Lineweaver–Burk and Michaelis–Menten plots showed that Pt-Tyr NZ had a higher affinity (lower *K*_M_ values) for both H_2_O_2_ and TMB compared to HRP, confirming its superior catalytic efficiency. The peroxidase activity of Pt-Tyr NZ was further validated through time-dependent absorbance studies, where TMB oxidation produced TMB^+^ (blue color) and TMB^2+^ (yellow color) upon stop solution addition. The interaction of cDNA with the Pt-Tyr NZ surface passivated its active sites, decreasing catalytic activity, while hybridization with miRNA-21 restored peroxidase activity by detaching cDNA. A calibration curve for miRNA-21 detection demonstrated an LDR of 0.00–185 nM, an LOD of 11.1 nM, and an LOQ of 36.9 nM, with excellent reproducibility (RSD < 2%). In selectivity studies, interfering RNAs showed negligible effects on Pt-Tyr NZ peroxidase activity. Finally, in 10% FHS samples, miRNA-21 was accurately determined in spiked samples with a recovery rate of 109.7 ± 4.2%, highlighting the potential of the Pt-Tyr NZ-based biosensing system for practical applications in clinical cancer diagnostics. The developed method is a one-step, easy-to-operate approach that can be used in clinical analysis as a diagnostic kit for the rapid quantification of miRNA-21.

## Supplementary Material



## Notes

The remaining information for Section[Sec sec2.4] (Catalytic Activity Determination), including preliminary testing of the spectrophotometer’s precision, optimization and determination of the catalytic activity of Pt-Tyr NZ/HRP, inhibition of peroxidase activity by cDNA-functionalized Pt-Tyr NZ, and spectrophotometric detection of miRNA-21; the oligonucleotides used in this study (Table S1); Figures S1–S6 (PDF)
