# Supplementary material for: Tyrosine-Capped Pt Nanozyme Functionalized with cDNA: An Innovative Sensor Designed for One-Step Detection of the miRNA-21 Biomarker
Source: ACS Omega. 2025 Aug 24;10(35):40324–34. doi: 10.1021/acsomega.5c05564 (PMC12423885; doi:10.1021/acsomega.5c05564)
Supplement: Supplementary file 1 [file ao5c05564_si_001.pdf]

Supplementary Data

Tyrosine-capped Pt nanozyme functionalized with cDNA: An innovative sensor designed for one-step detection of miRNA-21 biomarker

Sanam Garehbaghi*[a], Lukas Richtera[b], Zeynep Altintas[c,d], Amir M. Ashrafi*[e]

[a]Central European Institute of Technology, Brno University of Technology, 612 00, Brno, Czech Republic

[b]Department of Chemistry and Biochemistry, Mendel University in Brno, 613 00, Brno, Czech Republic

[c]Bioinspired Materials and Biosensor Technologies, Institute of Materials Science, Faculty of Engineering, Kiel University, 24143, Kiel, Germany

[d]Kiel Nano, Surface and Interface Science (KiNSIS), Kiel University, 24118, Kiel, Germany

[e]Institute of Photonics and Electronics, Czech Academy of Sciences, 182 00, Prague, Czech Republic

1. Catalytic activity determination

1.1. Preliminary test for preciseness of spectrophotometry device

For detecting preciseness of this spectrophotometry device, a 4 mM aqueous solution of potassium hexacyanoferrate(II) trihydrate which has an absorbance peak at the wavelength of 415 nm. The amount of 245 μL of solution was transferred to each cell of 96 well plate. The average absorbance for one cell at 5 repeats was 2.093 ± 0.003. The average absorbance among different rows of cell in one column and for different columns of cell in one row, were 2.091 ± 0.009 and 2.059 ± 0.029 respectively. Furthermore, the same series of experiments was carried out with 40 μM Methylene blue of 245 μL with a peak at wavelength of 660 nm with an absorbance nearly 1.5 absorbance was prepared in ultrapure water and transferred to each cell of 96 well plate. The average absorbance for different rows of one column and for different columns of one row were 1.545 ± 0.012 and 1.543 ± 0.016, respectivelly. While the average absorbance for one cell at 5 repeats was 1.559 ± 0.003. These results indicate a small random error in spectrophotometric measurement and pipetting and the absorbance value differences higher than 3σ (about 0.09) can be attributed to analyte.

1.2. Catalytic activity optimization and determination of HRP/Pt-Tyr NZ

The oxidase and peroxidase catalytic activity of 2.00 mg L^{-1} Pt-Tyr NZ were measured in 0.05 M PBS, with substrate solution (containing 0.8 mM H_2O_2 and 0.4 mM TMB) where after 8 min of reaction time 50 μL molecular biology grade (MBG) was added, and absorbance was read at wavelength 650 nm versus 500 nm in four repeats. Additionally, the peroxidase catalytic activity of 2.00 mg L^{-1} Pt-Tyr NZ

was investigated in 0.05 M PBS and substrate solution at various time periods after adding a stop solution containing 50 µL H_2SO_4 (0.5 M) to stop the reaction and reaching a final measurement solution of 245 µL. After adding the stop solution, the peroxidase activity was read at wavelength 450 nm versus 550 nm. Additionally, the catalytic activity for different concentrations of Pt-Tyr NZ in four repeats was measured in 0.5 M PBS solution after adding substrate solution followed by stop solution addition after 8 min.

For comparing the peroxidase-like activity of HRP and Pt-Tyr NZ, two substrate solutions initially were prepared as follows: solution I containing H_2O_2 (0.80 mM), and TMB (0.00, 0.16, 0.24, 0.32, 0.40, and 0.48 mM) in 0.05 M PBS, and solution II containing H_2O_2 (0.00, 0.25, 0.50, 0.75, 1.00, 1.25, and 1.50 mM), and TMB (0.50 mM) in 0.05 M PBS. In the next step, the Pt-Tyr NZ (2.00 mg L^{-1}) or HRP (0.004 mg L^{-1}) catalytic activity towards TMB in the solution I, and the Pt-Tyr NZ (2.50 mg L^{-1}) and HRP (0.001 mg L^{-1}) catalytic activity towards H_2O_2 in the solution II were assessed at wavelength 650 nm versus 500 nm using spectrophotometry for kinetic studies in four measurements. To efficiently compare the kinetic parameters of HRP and Pt-Tyr NZs, the catalytic activity assays were performed at similar condition (pH 7.4 and room temperature) in 0.05 M PBS solution.

After the conversion of absorbance to reaction velocity using the Beer-Lambert law (Eq. 1), the Michaelis-Menten (MM) plot[1] was drawn and a Lineweaver-Burk curve was utilized to assess K_M and V_{max} through transforming the MM equation into a double reciprocal linearization.[2]

In evaluating kinetic parameters based on the MM equation (Eq. 2), K_M is Michaelis constant and represents the affinity between the HRP/Pt-Tyr NZ and substrate (i.e., TMB and H_2O_2), and V_{max} is the maximal reaction velocity at a saturated concentration of substrate(s):[3]

$$v = \frac{\Delta A}{\Delta t \times \varepsilon \times l} \qquad (1)$$

$$v = \frac{(V\text{max} \times [\text{S}])}{K\text{M}} + V\text{max} \qquad (2)$$

where v is the reaction rate (µM min^{-1}), [S] is the substrate concentration (mM), V_{max} is the maximal catalytic velocity (µM min^{-1}), ΔA is the change in absorbance, Δt is the change in time (min), ε is the absorbance coefficient of TMB^+ (39,000 M^{-1} cm^{-1}), and l is the length of light through the well (cm).

The turnover number *(k_{cat})* for the HRP/Pt-Tyr NZ-catalyzed reaction was also estimated as follows (Eq. 3):[4]

$$K_{cat} = \frac{V_{max}}{[E]} \qquad (3)$$

where $[E]$ is the concentration of Pt-Tyr NZ or HRP.

1.3. *cDNA functionalized peroxidase activity-inhibiting of Pt-Tyr NZ*

This experiment was carried out according to the method in previous studies[5, 6] with minor modifications. In brief, Pt-Tyr NZ was incubated for 10 min with various concentrations of cDNA diluted in 40 µL MBG water to produce cDNA conjugated Pt-Tyr NZ. Subsequently, peroxidase reaction occurred in 0.05 M PBS and substrate solution, and reaction-termination was conducted by adding stop solution after 8 min. In 245 µL measurement solution the suppression of the peroxidase activity of 2.00 mg L^{-1} Pt-Tyr NZ was monitored when conjugating in the presence of different concentrations of cDNA (185, 371, 556, 742, 927 nM) with 4 times measurement.

1.4. *Spectrophotometric sensing of miRNA-21*

2.00 mg L^{-1} Pt-Tyr NZ conjugated with 556 mM cDNA was used for miRNA-21 assay in 245 µL measurement solution. Pt-Tyr NZ conjugated with cDNA was incubated with various concentrations of miRNA-21 in PBS solution incubating at room temperature for 30 min for the hybridization of cDNA to miRNA-21. To begin the peroxidase reaction, substrate solution and stop solution (after 8 min) were added sequentially and the absorbance was measured. The relative standard deviation (RSD) was calculated for repetitive measurements and reported as the reproducibility when six different solutions were measured and as the repeatability when one sample solution was measured five times. To examine the selectivity of the developed approach towards 74.2 nM miRNA-21, two miRNA cancer biomarkers of miR-17-3p and hsa-miR-1268b and one random RNA all at concentrations of 74.2 nM were used.

The HS sample was initially filtered through a 10 kDa Amicon ultra centrifugal filter (0.5 mL) using a 5424 R Eppendorf centrifuge (Eppendorf, San Diego, CA, USA) at 9391 ×g for 10 min at 4 °C.[7] Then, the resulting supernatant was diluted in a 0.05 M PBS to obtain a 10% (v/v) FHS. The different concentrations of miRNA-21 (18.6, 37.1, 74.2, 111, 148 nM) were then spiked into the mixture of 2.00 mg L^{-1} Pt-Tyr NZ conjugated with 556 mM cDNA in 10% FHS. After a 30 min incubation, a substrate solution was added and later the TMB's oxidation reaction was stopped by adding a stop solution after 16 min reaching a total measurement solution of 245 µL. The recovery rate and RSD were calculated according to the standard addition method.

Table S1. A list of oligonucleotides with different sequences

**Oligonucleotide**	**Sequence**
miR-17-3p	ACUGCAGUGAAGGCACUUGUAG
miRNA-21	UAGCUUAUCAGACUGAUGUUGA
cDNA	TCAACATCAGTCTGATAAGCTA
Random RNA	CGGUCGUGGCGGUGGGGGUA
hsa-miR-1268b	CGGGCGUGGUGGUGGGGGUG

5. Supporting Figures:

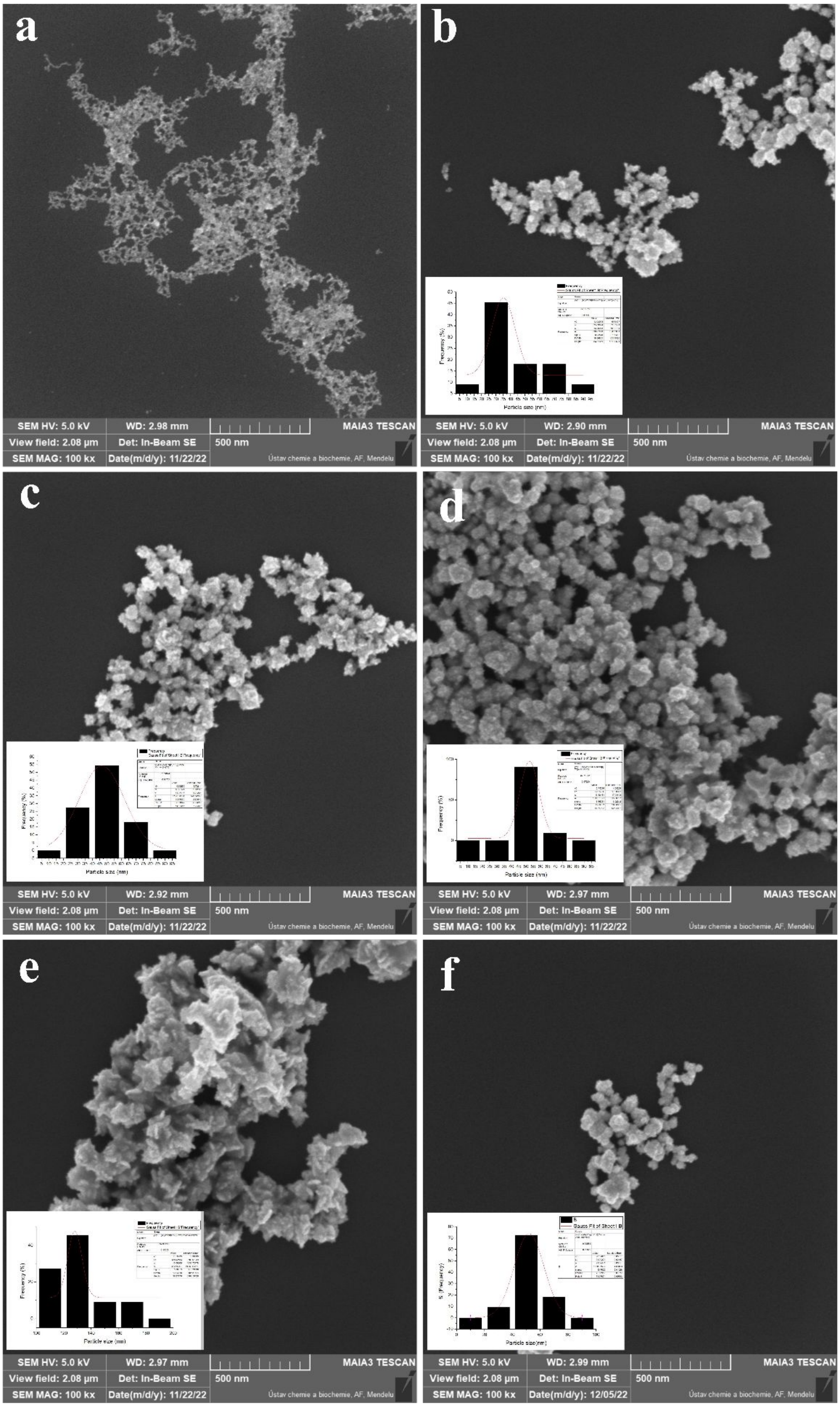

Figure S1. SEM images for Pt NZs synthesized with Tyr amounts of 0.00 (a), 5.00 (b), 10.0 (c), 15.0 (d), and 20.0 (e) mg in the aqueous synthesis media. SEM image of Pt-Tyr after modification with cDNA (f).

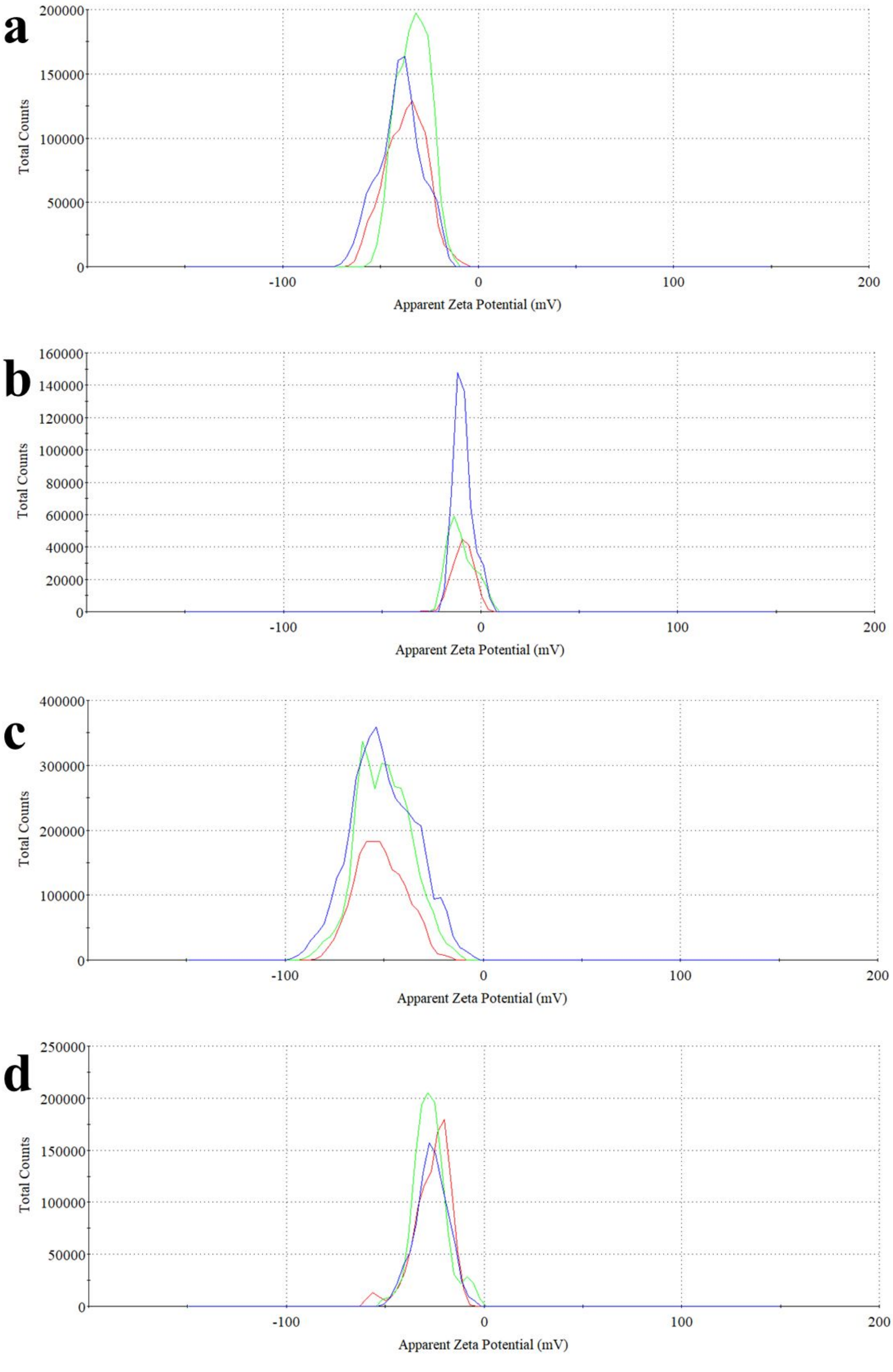

Figure S2. ζ-potential for Pt-Tyr NZ before dialysis (a), Pt-Tyr NZ after dialysis (b), Pt-Tyr NZ conjugated with cDNA (c), Pt-Tyr NZ conjugated cDNA after introducing miRNA-21(d)- in three repeats.

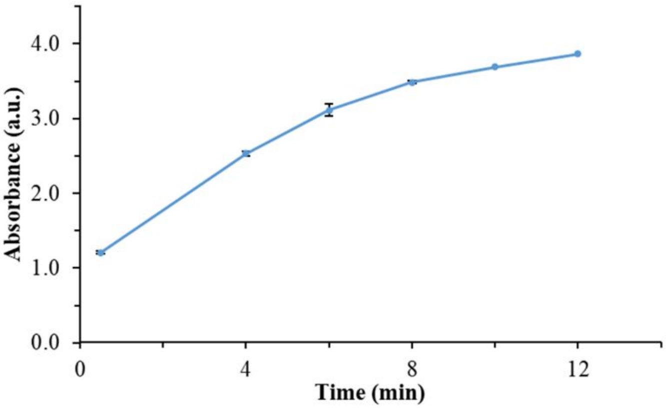

Figure S3. The time-dependent peroxidase activity of Pt-Tyr NZ determined by absorbance intensity at wavelength 450 nm after adding stop solution in various reaction times.

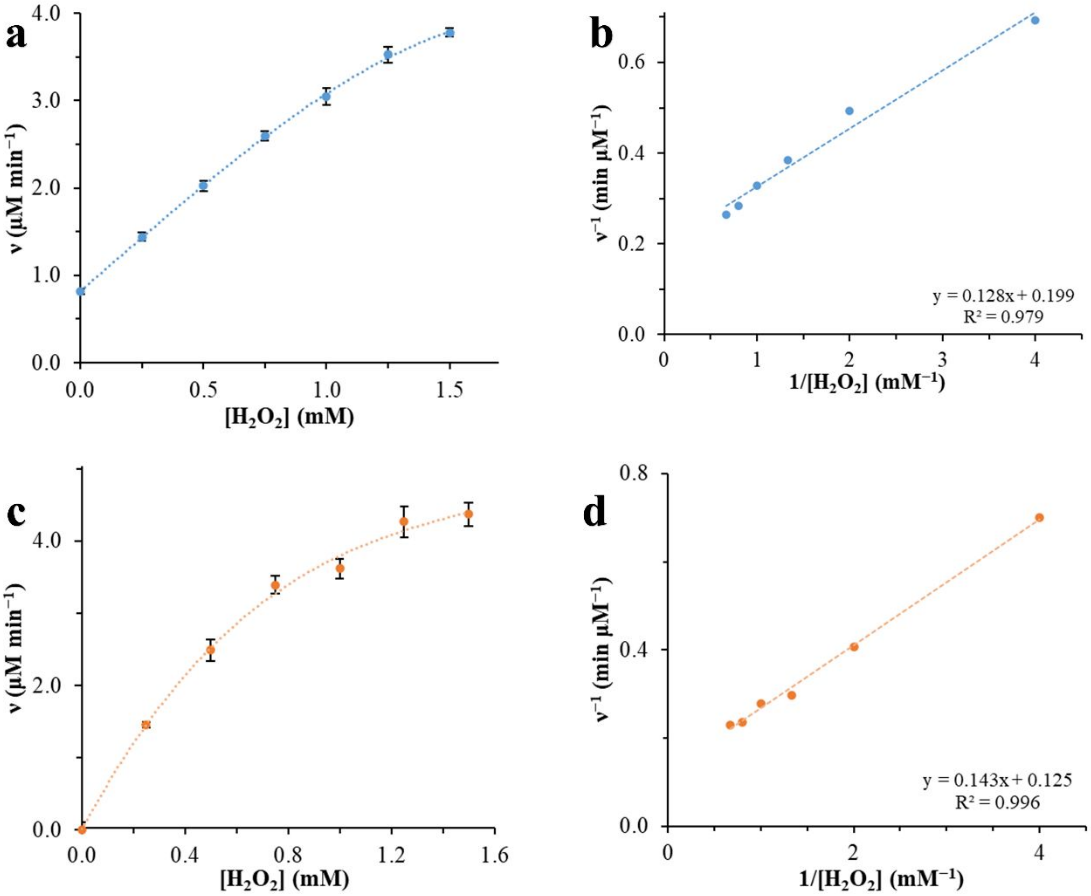

Figure S4. MM (a) and Linweaver-Burck (b) curves for 2.50 mg L^{-1} Pt-Tyr NZ. MM (c) and Linweaver-Burck (d) curves for 0.001 mg L^{-1} HRP towards H_2O_2.

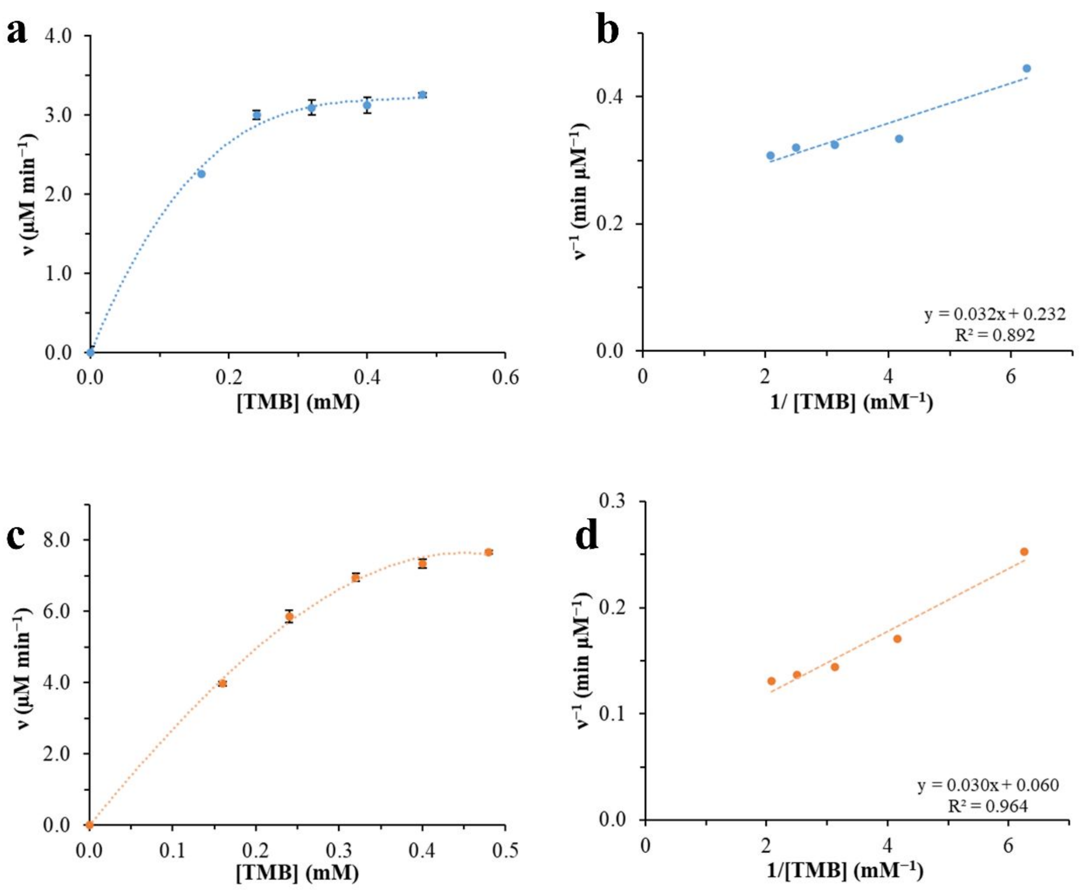

Figure S5. MM (a) and Linweaver-Burck curves (b) for 2.00 mg L^{-1} Pt-Tyr NZ. MM (c) and Linweaver-Burck curves (d) for 0.004 mg L^{-1} HRP towards TMB.

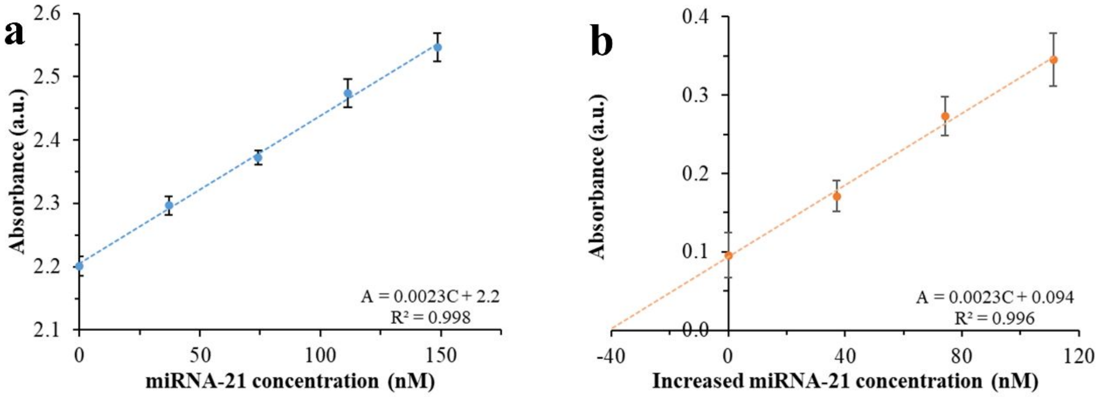

Figure S6. Calibration (a) and standard addition (b) curves for the detection of miRNA-21 in 10% FHS samples.

References

(1) Wang, H.; Zhao, J.; Liu, C.; Tong, Y.; He, W. Pt Nanoparticles Confined by Zirconium Metal-Organic Frameworks with Enhanced Enzyme-like Activity for Glucose Detection. *ACS omega* **2021**, *6*, 4807-4815.

(2) Mikysek, T.; Frühbauerová, M.; Švancara, I.; Novák, M.; Sýs, M. A New Voltammetric Approach for the Determination of Biomimetic Catalyst Kinetic Constants Based on Substrate Consumption. *Electroanalysis* **2023**, *35*, e202200269.

(3) He, J.; Yang, L.; Zhang, Y.; Li, R.; Wu, J.; Cao, Q.; Li, S.; Wu, X.; Duan, Y.; He, D.; Wang, W. Pd–Pt–Ru nanozyme with peroxidase-like activity for the detection of total antioxidant capacity. *Anal. Methods* **2023**, *15*, 8-16.

(4) Dusíková, A.; Baranová, T.; Krahulec, J.; Dakošová, O.; Híveš, J.; Naumowicz, M.; Gál, M. Electrochemical Impedance Spectroscopy for the Sensing of the Kinetic Parameters of Engineered Enzymes. *Sensors* **2024**, *24*, 2643.

(5) Huang, L.; Chen, K.; Zhang, W.; Zhu, W.; Liu, X.; Wang, J.; Wang, R.; Hu, N.; Suo, Y.; Wang, J. ssDNA-tailorable oxidase-mimicking activity of spinel MnCo2O4 for sensitive biomolecular detection in food sample. *Sens. Actuators B: Chem.* **2018**, *269*, 79-87.

(6) Chen, W.; Fang, X.; Ye, X.; Wang, X.; Kong, J. Colorimetric DNA assay by exploiting the DNA-controlled peroxidase mimicking activity of mesoporous silica loaded with platinum nanoparticles. *Microchim. Acta* **2018**, *185*, 544.

(7) You, R.; Li, J.; Wang, H.; Wu, Y.; Weng, J.; Lu, Y. High-performance SERS biosensor based on in-situ reduction of silver nanoparticles in an ultra-filtration centrifuge device for label-free detection of colon cancer in serum. *J. Membr. Sci. Res.* **2023**, *678*, 121688.
